# Meandering Mesenteric Bypass: A Case Report on a Novel Surgical Technique for Managing Mesenteric Ischaemia When Endovascular Fails

**DOI:** 10.1016/j.ejvsvf.2024.10.002

**Published:** 2024-10-05

**Authors:** Geoffrey Ying, Andrew Hill, Sam Taylor, Anastasia Dean

**Affiliations:** a University of Auckland, Faculty of Medical and Health Sciences, Auckland, New Zealand; b Auckland Regional Vascular Service, Auckland City Hospital, Auckland, New Zealand

**Keywords:** Acute on chronic mesenteric ischaemia, Aorto-iliac disease, Case report, Distal embolisation, Endovascular revascularisation, Mesenteric collateral vessel bypass

## Abstract

**Introduction:**

Endovascular revascularisation (ER) is often used as first line treatment for chronic mesenteric ischaemia, with high technical success and a lower rate of peri-operative adverse events than open surgical repair. Distal embolisation following ER is a potentially life threatening complication with a high mortality rate.

**Report:**

A 66 year old patient with a two year history of postprandial abdominal pain presented with two weeks of constant abdominal pain. Computed tomography angiography (CTA) demonstrated extensive atherosclerotic disease within the coeliac artery (CA), superior mesenteric artery (SMA), inferior mesenteric artery (IMA), aorta, and iliac arteries. Stenting of the CA was successful. Four hours post-intervention, the patient became unstable with worsening abdominal pain. Repeat CTA showed signs of acute bowel ischaemia (pneumatosis, mural hypo-enhancement, and intravascular gas) despite a patent CA stent. Microemboli trashing secondary to ER was suspected.

The patient was taken for an emergency laparotomy. The SMA was an unsuitable bypass target due to extensive atherosclerosis, and bypass to the CA was unlikely to improve perfusion, given the patent stent. A large, disease free collateral artery was identified as a potential bypass target, and a six mm Dacron graft was successfully anastomosed from the right external iliac artery to the Arc of Riolan. At the relook laparotomy, the iliac–mesenteric bypass graft was covered and the abdomen was closed. The patient has since had complete resolution of symptoms and 12 kg of weight gain at six months.

**Discussion:**

This is a unique case in which the Arc of Riolan was used as a bypass target in the context of acute on chronic mesenteric ischaemia. It highlights the possibility of collateral mesenteric vessels as potential bypass targets when the mesenteric trunks are unsuitable. Good knowledge of collateral vessel anatomy and careful pre-operative planning is critical in patients with extensive disease that is not amenable to traditional treatment.

## HIGHLIGHTS

• Distal embolisation is a complication of mesenteric endovascular revascularisation with high morbidity and mortality rates.
• Extensive calcification and aorto–iliac disease often limit the options for open surgical bypass.
• The meandering mesenteric artery can serve as a bypass target when the mesenteric trunks are unsuitable.

## INTRODUCTION

Endovascular revascularisation (ER) is a rapidly expanding field in the treatment of chronic mesenteric ischaemia (CMI), with high technical success and a lower rate of peri-operative adverse events (AE) than open surgical repair. While long term outcomes for recurrence and need for re-intervention are not as favourable as in open repair, ER makes for an attractive alternative, especially in higher risk patients. Consequently, the number of endovascular procedures has increased ten fold over the last decade and now accounts for >70% of initial revascularisation procedures.^1^^,^^2^ The most common procedure related AEs of endovascular intervention are access related and distal embolisation, which can lead to acute mesenteric ischaemia (AMI) and bowel necrosis.^3^^,^^4^

There is minimal literature describing the management of AMI secondary to mesenteric angioplasty and stenting. Treatment usually involves urgent revascularisation and resection of necrotic bowel. However, factors such as location of blockage, suitability of inflow and target vessels for bypass, patient condition, and previous interventions often limit the feasibility of standard revascularisation techniques. This case report describes a novel approach where the Arc of Riolan was used as a bypass target in the setting of acute on chronic mesenteric ischaemia precipitated by prior endovascular intervention. Informed consent for publication was obtained from the patient.

## REPORT

**Figure 1:**
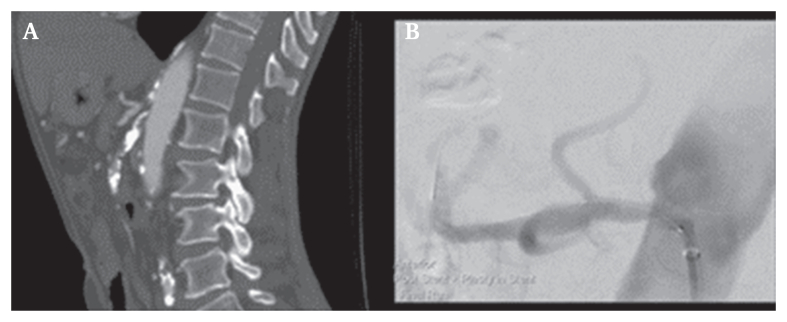
Computed tomography angiography images. A. Stenosis of the coeliac artery and occlusion of the superior mesenteric artery. B. Completion run post-coeliac artery stenting.

A 66 year old patient presented to the emergency department with a two week history of abdominal pain on a background of postprandial abdominal pain for the past two years. The pain was localised to the epigastric and umbilical regions and associated with 10 kg of weight loss. The patient denied fever, rectal bleeding, nausea and vomiting, and his bowels were regular. His cardiovascular risk factors included hypertension, dyslipidaemia, and he was an ex-smoker with a 30 pack year history. He had no prior investigations or intervention for mesenteric ischaemia. Physical examination identified generalised abdominal tenderness without signs of peritonism. Investigations on admission showed an elevated white cell count (14 x 10^9^/L), elevated C reactive protein (130 mg/L), and a normal blood lactate (0.8 mmol/L). Computed tomography angiography (CTA) demonstrated critical stenosis of the coeliac artery (CA), a long occlusion of the SMA, and extensive atherosclerosis within the inferior mesenteric artery, aorta, and iliac arteries (Fig. 1A). The gastroduodenal artery was patent but small in calibre; the collaterals between the SMA and IMA were well developed. Gastroscopy was unremarkable.

The SMA was not amenable to endovascular, open, or hybrid revascularisation due to a long flush occlusion and extensive calcification continuing distally into the branches. Consequently, attention was turned to the CA, which had a focal stenosis with a relatively disease free artery beyond the lesion. The IMA was patent and intervention here was unlikely to produce benefit.

From a femoral approach, the CA was pre-dilatated with a 3 mm semi-compliant balloon, followed by successful deployment of a 5 x 18 mm drug eluting balloon expandable stent. Given the successful CA intervention, no attempt was made to recanalise the SMA. Completion imaging showed improved perfusion of the coeliac artery branches and no residual stenosis (Fig. 1B).

**Figure 2:**
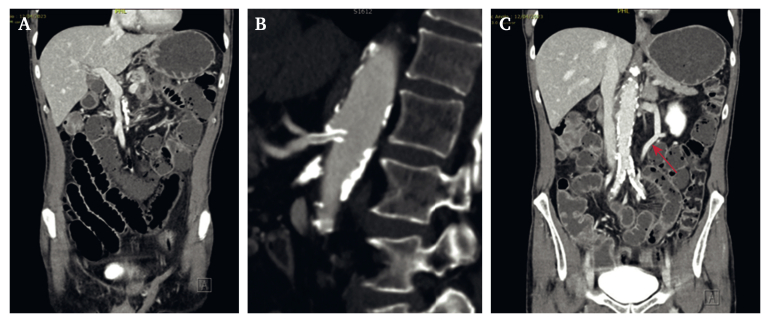
Repeat computed tomography angiography images. A. Ischaemic changes. B. Patent coeliac artery stent. C. Arc of Riolan (indicated with red arrow).

Four hours post-intervention, the patient developed worsening abdominal pain and became haemodynamically unstable, with tachycardia and hypertension. The abdomen was tender without signs of peritonism. Repeat blood tests showed an elevated white cell count (18 x 10^9^/L). A repeat CTA showed dilated small bowel loops with pneumatosis and mural hypo-enhancement, and gas in the small bowel mesenteric vessels and peripheral intrahepatic portal veins, all of which was concerning for acute small bowel ischaemia (Fig. 2A). The coeliac artery stent was patent and there was no flow deficit on imaging (Fig. 2B). Clinical deterioration was suspected to be due to athero-embolisation from wire and catheter manipulation in the diseased aorta or CA.

Given the patient's rapid deterioration and need to assess bowel viability, repeat endovascular intervention with thrombolysis was not appropriate. The SMA and IMA were both unsuitable bypass targets, given the length and extent of the atherosclerosis. Furthermore, it was decided that an open bypass to the CA or common hepatic artery would not be beneficial given that the stent was still patent. A large, disease free collateral artery (the Arc of Riolan) was identified as a potential target if the bowel was salvageable (Fig. 2C).

**Figure 3:**
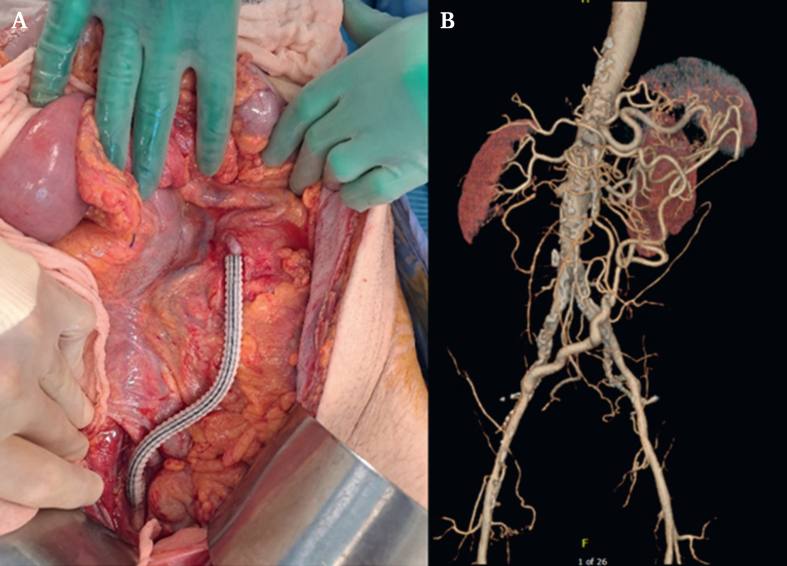
Bypass graft. A. Intra-operative image of the bypass graft. B. Graft surveillance six months after surgery.

At midline laparotomy, the proximal jejunum was dusky, but there was no frank necrosis or faecal contamination. A large, tortuous collateral artery was identified in the left infra-colic compartment extending superiorly into the transverse mesocolon. Flow through the Arc of Riolan arcade was assessed via palpation (weakly pulsatile) and continuous Doppler ultrasound (biphasic flow). The right external iliac artery was found to be a satisfactory donor vessel. A 6 mm Dacron graft was anastomosed from the right external iliac artery to the Arc of Riolan, both in an end to side fashion (Fig. 3A). A Dacron graft was used as the field was not contaminated and the patient did not have a suitable calibre vein. The abdomen was closed with a temporary abdominal closure system in preparation for second look surgery in 48 hours. The patient continued on antibiotics. At the second look laparotomy, the bowel was viable with minor serosal ulceration, which was repaired. The Dacron iliac–mesenteric bypass graft was covered with an omental patch, and the abdomen was closed. Post-operatively, the patient was admitted to the intensive care unit. He was transferred to the ward after two days, given resolution of symptoms, clinical stability, and down trending inflammatory markers. He was discharged two weeks following the operation, and had gained 12 kg of weight at his six month follow up. Computed tomography angiography surveillance showed that the graft was satisfactory but has developed some tortuosity (Fig. 3B).

## DISCUSSION

Distal embolisation following ER for CMI is a life threatening complication with a high mortality rate.^4^^,^^5^ Endovascular manipulation can disrupt calcified atherosclerotic plaque and intraluminal thrombus. This can lead to bowel infarction, which often necessitates rescue intervention such as open revascularisation and bowel resection, which is associated with a poor prognosis.^4^^,^^6^ Distal embolisation, especially microemboli trashing, can be missed on completion angiography, leading to delay in diagnosis and treatment with potentially disastrous consequences.^5^, ^6^, ^7^ It is suspected that endovascular manipulation in the diseased aorta and visceral arteries may have resulted in distal embolisation via mesenteric collaterals, leading to clinically significant acute on chronic mesenteric ischaemia.

The rate of distal embolisation during endovascular revascularisation is estimated at 0–11% in the literature.^5^^,^^8^ Complete vessel occlusion, severe calcification, and lesion length > 30 mm were factors associated with a greater risk of distal embolisation.^8^ Prompt intervention with restoration of intestinal perfusion is critical to improving prognosis. Rescue treatment options include further stenting in the setting of a dissection, catheter directed thrombolysis or thrombus aspiration, open embolectomy, and surgical bypass.^5^^,^^9^

In this case, further stenting was unlikely to be of benefit as the previously placed coeliac stent was still patent with no dissection. Similarly, catheter directed thrombolysis or suction thrombectomy was not preferred as there was no obstruction visible on imaging, and rapid clinical deterioration and radiological signs of bowel ischaemia necessitated an emergency laparotomy. The SMA is typically recommended as the primary target for open bypass in the setting of mesenteric ischaemia,^1^^,^^2^ with the CA and IMA as viable alternatives. However, bypass to the SMA was not feasible, given the occlusive atherosclerosis extending into the branches. The IMA was similarly diseased. Surgical bypass to the CA or common hepatic artery was thought unlikely to improve overall perfusion as the primary problem was in the midgut and the coeliac stent was patent. As such, the collateral between the SMA and IMA was an attractive target. With limited options, the Arc of Riolan was used to maximise perfusion to the branches of both the IMA and SMA, with the aim of bypassing potential blockages caused by the microemboli trashing. Its large calibre made it relatively easy to access with minimal dissection. The external iliac artery was the optimal inflow artery, given the extent of atherosclerosis in the patient's aorta.

Testart *et al.* reported a similar revascularisation procedure for two patients with CMI and intestinal necrosis by anastomosis of the left renal artery to the marginal artery of Drummond.^10^ This was performed in a similar setting of CA and SMA occlusion, although the rationale was to avoid cross clamping the aorta and entering an infected peritoneal cavity.

This was a unique case in which the Arc of Riolan was used as a bypass target in the setting of acute mesenteric ischaemia. It highlights the possibility of collateral mesenteric vessels as potential bypass targets when the mesenteric trunks are unsuitable. Good knowledge of collateral vessel anatomy and careful pre-operative planning to identify such vessels is critical in patients with extensive disease not amenable to traditional treatment. Prompt identification of clinical deterioration following endovascular revascularisation for CMI is vital to managing life threatening complications. Deteriorating patients often require open surgery to assess bowel viability. Open surgical bypass remains a key revascularisation strategy.

## FUNDING

This research did not receive any specific grant from funding agencies in the public, commercial, or not for profit sectors.
